# Antimicrobial Susceptibility Profiles and Molecular Characterisation of *Staphylococcus aureus* from Pigs and Workers at Farms and Abattoirs in Zambia

**DOI:** 10.3390/antibiotics11070844

**Published:** 2022-06-24

**Authors:** Mulemba Tillika Samutela, Bruno Stephen July Phiri, Edgar Simulundu, Geoffrey Kwenda, Ladslav Moonga, Eugene C. Bwalya, Walter Muleya, Therese Nyirahabimana, Kaunda Yamba, Henson Kainga, Simegnew Adugna Kallu, Innocent Mwape, Andrew Frey, Matthew Bates, Hideaki Higashi, Bernard Mudenda Hang'ombe

**Affiliations:** 1 Department of Biomedical Sciences, School of Health Sciences, University of Zambia, Lusaka 10101, Zambia; jaffekwenda@gmail.com; 2 Department of Paraclinical Studies, School of Veterinary Medicine, University of Zambia, Lusaka 10101, Zambia; ladslav.moonga@unza.zm (L.M.); bhangombe@unza.zm (B.M.H.); 3 Central Veterinary Research Institute, Lusaka 10101, Zambia; julypondayapa@yahoo.com; 4 Department of Disease Control, School of Veterinary Medicine, University of Zambia, Lusaka 10101, Zambia; esikabala@yahoo.com (E.S.); kaundayamba@gmail.com (K.Y.); hkainga@bunda.luanar.mw (H.K.); adusim12@gmail.com (S.A.K.); 5 Macha Research Trust, Choma P.O. Box 630166, Zambia; 6 Department of Clinical Studies, School of Veterinary Medicine, University of Zambia, Lusaka 10101, Zambia; eugene.bwalya@unza.zm; 7 Department of Biomedical Sciences, School of Veterinary Medicine, University of Zambia, Lusaka 10101, Zambia; muleyawalter@gmail.com (W.M.); ntherese22@gmail.com (T.N.); 8 Department of Pathology and Microbiology, University Teaching Hospitals, Lusaka 10101, Zambia; 9 Department of Veterinary Epidemiology and Public Health, Faculty of Veterinary Medicine, Lilongwe University of Agriculture and Natural Resources, Lilongwe 207203, Malawi; 10 College of Veterinary Medicine, Haramaya University, Dire Dawa P.O. Box 138, Ethiopia; 11 Center for Infectious Disease Research Zambia, Lusaka 10101, Zambia; innocent.mwape@cidrz.org; 12 Department of Cell Biology, Microbiology and Molecular Biology, University of South Florida, Tampa, FL 33620, USA; andrew.m.frey@gmail.com; 13 School of Life & Environmental Sciences, University of Lincoln, Lincolnshire LN6 7TS, UK; mbates@lincoln.ac.uk; 14 Division of Infection and Immunity, International Institute for Zoonosis Control, Hokkaido University, Sapporo 001-0020, Japan; hidea-hi@czc.hokudai.ac.jp

**Keywords:** antimicrobial resistance, *Staphylococcus aureus*, *spa* typing, swine, Zambia

## Abstract

Pigs have been shown to be a reservoir for recently emerging livestock-associated *Staphylococcus aureus* (LA-SA), including methicillin resistant strains in many countries worldwide. However, there is sparse information about LA-SA strains circulating in Zambia. This study investigated the prevalence, phenotypic and genotypic characteristics of *S. aureus* from pigs and workers at farms and abattoirs handling pigs in Lusaka Province of Zambia. A total of 492 nasal pig swabs, 53 hand and 53 nasal human swabs were collected from farms and abattoirs in selected districts. Standard microbiological methods were used to isolate and determine antimicrobial susceptibility patterns of *S. aureus*. Polymerase Chain Reaction was used to confirm the species identity and detect antimicrobial resistance and virulence genes of isolates, whereas genetic diversity was evaluated using *spa* typing. Overall prevalence of *S. aureus* was 33.1%, 37.8% for pigs and 11.8% for humans. The isolates were resistant to several antibiotics with resistance ranging from 18% to 98% but were all susceptible to vancomycin. Typical LA-SA *spa* types were detected. The presence of plasmid mediated resistance genes such as *tet*M (12.8%), other resistance determinants and immune evasion cluster genes among the isolates is of great public health concern. Thus, continuous surveillance of *S. aureus* using a “One health” approach is warranted to monitor *S.* *aureus* infections and spread of antimicrobial resistance.

## 1. Introduction

*Staphylococcus aureus*, a Gram-positive bacteria, is a pathobiont of humans and animals including pets, livestock and wildlife, with animal infections and reservoirs being a potential source for human infections and vice versa [1]. Currently, the epidemiology of *S. aureus* including methicillin resistant strains is classified into three hospital or healthcare-associated *S. aureus* (HA-SA and HA-MRSA, respectively), community-associated *S. aureus* (CA-SA) and livestock-associated *S. aureus* (LA-SA) [2]. *Spa* typing and multi-locus sequence typing (MLST) are widely used to derive *spa* types (t) and sequence types (STs) or clonal complexes (CCs), which have been determined globally. Some *spa* and ST types are commonly associated with LA-SA. Typically, LA-SA from pigs in Europe have been associated with CC398, whereas CC9 is the predominant type in Asia [3]. *Spa* types t011, t034, t108, t567, t571, t899, t1254, t1451, t2011 and t2510 are associated with CC398, and are among those more closely linked to LA-SA [4].

While a wide range of livestock are implicated with LA-SA, pigs are considered a major reservoir of these *S. aureus* strains [5]. Notably, the LA-SA have been detected in persons with occupational contact with pigs including farm and slaughterhouse workers and veterinarians [6,7]. Additionally, LA-SA has been isolated in persons without occupational contact to the animals [8]. Therefore, LA-SA are a source of concern as they can be passed on from animals to humans and from humans to humans. While LA-SA were mostly associated with colonisation and minor infections, blood stream infections (invasive infections) with livestock-associated methicillin-resistant *S. aureus* (LA-MRSA) have been reported in Germany and Denmark [9,10]. This is worrisome as it shows that these strains are not only circulating in the community but are entering hospitals thereby blurring the distinctions between the epidemiological groups of *S. aureus*. Such transmission is of major concern, because while the use of antibiotics in farm animals such as pigs may select for antimicrobial resistance, LA-SA have generally been more susceptible to antibiotics compared to the HA-SA due to excessive use of antibiotics and or poor antibiotic stewardship in hospitals. Therefore, the entry of LA-SA into health care institutions may lead to these strains acquiring resistance which could be passed back into their communities of origin as adapted strains.

While emphasis is mainly placed on MRSA, methicillin susceptible *S. aureus* (MSSA) strains are equally important in the evolution of *S. aureus*. Using whole genome sequencing, it has been shown that LA-CC398-MRSA evolved from an ancestor which was a human-adapted HA-MSSA CC398 [11]. The CC398-MSSA ancestor could have acquired resistance to methicillin and tetracycline while losing the prophage that carries the immune evasion cluster genes (IEC). The IEC genes protect *S. aureus* against the immune system in humans [12]. The presence or absence of the IEC genes can indicate whether *S. aureus* strains are human or livestock-associated, respectively. Several studies from European countries show that the LA-CC398 MSSA have emerged as a subpopulation of causative agents of invasive infections in hospitals [13,14,15,16,17]. Of interest is a subset of the CC398 MSSA which is independent of livestock but is human adapted [15]. However, there is sparse information on this clade. Therefore, more studies are warranted to further understand such lineages. Furthermore, given the role of human and animal interactions in the emergence of such lineages, it is critical to conduct such studies in a comprehensive manner using a “One Health” approach. Despite heightened interest in the epidemiology of LA-SA across the globe, there is still a paucity of data on the prevalence and characteristics of pig related LA-SA on the African continent. A recent systematic review revealed that only 19 studies specifically reported on the prevalence or incidence, antimicrobial susceptibility profiles and genetic characteristics of pig-associated *S. aureus* in Africa between 2000 and 2019 [18].

Pig farming is an important economic activity in Zambia, with most pig farmers being smallholder farmers in the rural areas of the country. However, commercial pig farming has over the years become more common in the more urban parts of the country especially in Lusaka Province. This shift could entail an increase in antibiotic use in pig rearing establishments, which has been shown to be a risk factor for the emergence of MRSA. Zambia, similarly to many other countries, has reported the presence of *S. aureus* infections in the clinical settings including multidrug resistant strains of MRSA [18,19], in pets [20] and wildlife [21]. However, there is scarce information on LA-SA. Therefore, this study aimed to determine the prevalence, phenotypic and genotypic characteristics of *S. aureus* in pigs and workers from farms and abattoirs handling pigs in Lusaka Province of Zambia.

## 2. Results

### 2.1. Prevalence of S. aureus in Pigs and Humans in Lusaka Province

The overall prevalence of *S. aureus* was 33.1% (Table 1). In pigs and workers, the prevalence was 37.8% and 11.3%, respectively. The positivity rate in both nasal and hand swabs from humans was 11.3% (Table 1). Chilanga District showed the highest (66.4%) positivity rate among the three districts studied (Table 1). Notably, some pig samples yielded more than one *S. aureus* isolate (*n* = 27) (Supplementary Materials Table S1) and these isolates were not included in the calculation of the prevalence but were further characterized. When broken down by district, the positivity rate of human samples was 30.4% (7/23), 8.7% (4/46) and 2.7% (1/37) for Chilanga, Chongwe and Lusaka districts, respectively. Therefore, positivity rate among humans was higher in Chilanga district compared with Chongwe and Lusaka districts (*p* = 0.005).

With respect to study sites, the overall prevalence at farms and abattoirs was 27.2% and 65.9%, respectively. The prevalence at abattoirs was comparatively higher than that of farms (Table 2). Generally, the prevalence of *S. aureus* in pigs was significantly higher than in humans at both the farm and abattoir levels (Table 1 and Table 2). While the prevalence of *S. aureus* was high for both pigs and humans at medium and large-scale facilities, the prevalence of *S. aureus* in humans was low in small-scale farms (Table 2).

### 2.2. Antimicrobial Susceptibility Profiles and Antimicrobial Resistance Genes Detected in the S. aureus Isolates

**Figure 1:**
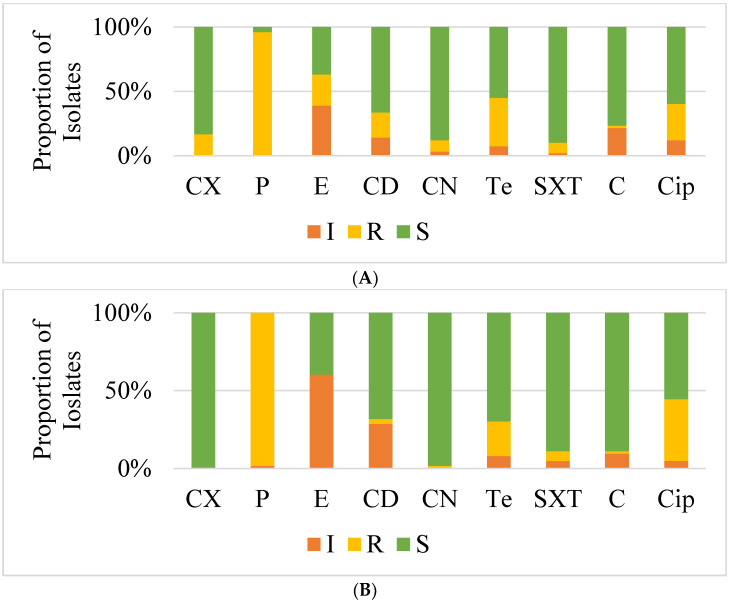
Overall Antimicrobial Susceptibility Profiles of *S. aureus* Isolates from pigs and workers from Farms (**A**) and Abattoirs (**B**) of Lusaka Province. Abbreviations: P = Penicillin; CN = Gentamicin; E = Erythromycin; CD = Clindamycin; Cip = Ciprofloxacin; Te = Tetracycline, SXT = Cotrimoxazole, C = Chloramphenicol, CX = Cefoxitin; I = Intermediate, R = Resistant, S = Susceptible.

The highest resistance of the *S. aureus* isolates from samples collected from both pigs and humans at the farms were to penicillin (98%), whereas resistance to tetracycline, ciprofloxacin and cefoxitin was recorded at 35%, 30% and 18%, respectively (Figure 1A). In addition, these isolates were more susceptible to co-trimoxazole (92%), gentamicin (90%) and chloramphenicol (79%) (Figure 1A). Forty percent of the isolates showed intermediate susceptibility to erythromycin, whereas erythromycin-induced clindamycin resistance was detected in only one isolate. Notably, all isolates tested against vancomycin were susceptible with minimum inhibition concentrations (MICs) ranging from 1.5 μg/L to 3 μg/L (Supplementary Materials Table S2). From abattoirs, the highest resistance was recorded to penicillin at 98% followed by 35% and 25% to ciprofloxacin and tetracycline, respectively (Figure 1B). From all isolates, 100% susceptibility was observed to cefoxitin, 99% to gentamicin, 90% to co-trimoxazole and 88% to chloramphenicol. Intermediate results were highest for erythromycin, whereas no erythromycin-induced clindamycin resistance was detected.

#### 2.2.1. Multidrug Resistance Patterns of the *S. aureus* Isolates

To assess whether antibiotic resistance phenotypes clustered together, antibiotic resistance patterns were assigned using designations PG + Te + CN + E + CD + Cip + C + SXT (as defined in Figure 1 legend). Isolates were grouped into 23 antibiotic resistance patterns (Table 3). A majority of the isolates were resistant to at least one or two antibiotics besides penicillin, with the predominant phenotype being P + Cip (15.7%) and P + E + CD + Cip (14.2%). Multi-drug resistance to a combination of three, four, five, and six antibiotics was observed in 17.6% of the isolates. Isolates were classified as multi-drug resistant (MDR) if, in addition to the Beta-lactams, they were resistant to 3 classes based on susceptibility to erythromycin, clindamycin, chloramphenicol, ciprofloxacin, tetracycline and co-trimoxazole. Based on this classification, 36 isolates from farms were MDR (Table 3).

#### 2.2.2. Presence of Antimicrobial Resistance Genes in the *S. aureus* Isolates

The *mecA* and *mecC* genes that encode for methicillin resistance were not detected in all the isolates despite the phenotypic resistance to methicillin in some of the isolates. With respect to the genes encoding for tetracycline resistance, detection rates were 19.3% (11/57) for *tet*M, 12.3% (7/57) for *tet*K and 1.8% (1/57) for *tet*L. Notably, all isolates harbouring these genes were from nasal pig swabs. The *tet*O gene was not detected in any of the isolates tested. Only one isolate harboured both *tet*M and *tet*L genes. Farm isolates harboured more of the tetracycline resistance genes than those from the abattoirs. The *erm*B and *erm*C genes were detected in 19.2% (5/26) and 57.7% (15/26) of the isolates, respectively, whereas *erm*A was not detected in any of the isolates. Most of the isolates harbouring these resistance genes were from pigs sampled from farms. Only two human isolates harboured the resistance genes. Notably, all isolates resistant to erythromycin were from the same farm.

### 2.3. Virulence Genes Detected in the S. aureus Isolates

Neither the Panton-Valentine Leukocidin (PVL) nor the staphylococcal enterotoxin (SE) encoding genes were detected in any isolates in this study. For the IEC genes, *sak*, *scn* and *chp*, were detected in 7.6% (17/225), 1.3% (3/225) and 0.4% (1/225) isolates, respectively (Table 4). All these isolates were from nasal swabs of pigs, mostly from one farm (Farm 7) (Table 4).

### 2.4. Spa Typing of the S. aureus Isolates

All *S. aureus* isolates (*n* = 225) were positive for the *spa* gene by PCR and 43 representative isolates based on the most frequent resistance phenotypes were sequenced to determine the *spa* types (Supplementary Materials Table S3). Six *spa* types were detected namely, t1430 (*n* = 12), t034 (*n* = 8), t318 (*n* = 4), t571 (*n* = 1), t084 (*n* = 1) and t899 (*n* = 1). The most common *spa* type was t1430 (28.0%) followed by t034 (18.6%) (Table 5). Only *spa* type t1430 was detected in both humans and pigs, *spa* types t034, t318, t571 were found in pigs only while t084 and t899 were found in humans only (Table 5). A total of 16/43 (37.3%) of the isolates were of unknown *spa* types (Table 5). The two most common *spa* types, t1430 and t034, were found at both farms and abattoirs of medium and large scale from all the three districts (Supplementary Materials Table S3). Notably, t1430 was detected in most of the nasal pig swabs and one human hand swab at abattoir 1 (Supplementary Table S3). The isolates with unknown *spa* types were mostly from medium scale facilities (Supplementary Materials Table S3).

## 3. Discussion

This study aimed at determining the prevalence, phenotypic and molecular characteristics of *S. aureus* from pigs and workers from pig farms and abattoirs in the Lusaka Province of Zambia. This is the first report on the presence of *S. aureus* in pigs and workers from farms and abattoirs in Zambia. The overall prevalence rate (33.1%) of *S. aureus* in the present study was relatively high and is in congruence with similar studies on the African continent that have reported prevalences ranging from 0% to 55% [18]. However, specific comparisons of prevalences is difficult due to the variations in the conduct of these studies, for example, most studies have only studied isolates either from farms or abattoirs and not from both sites [1]. In addition, some studies may sample from more than one body part of the pigs [1]. A comparatively higher prevalence of *S. aureus* was detected in the pigs (37.8%) than in workers (11.3%), similar to the findings from a recent study in Nigeria [22]. However, the studies from Nigeria and South Africa detected more *S. aureus* from pigs than in our study [22,23]. The current study further showed that hand and nasal prevalence of *S. aureus* was the same among workers.

The antimicrobial susceptibility profiles of the isolates revealed that most isolates from farms and abattoirs were resistant to several antibiotics, with the highest resistance being to penicillin (98%). This finding is significantly higher than that from the study in Nigeria which reported a lower resistance to penicillin of 55% [22]. The high resistance to penicillin reflects possible overuse of the antibiotic, as penicillin is generally among most frequently used antibiotics in many farms in many countries [22,24,25]. Resistance to tetracycline, erythromycin and ciprofloxacin was also recorded in 25% to 35% of isolates in our present study. Notably, tetracycline is also commonly used to treat infections in both humans and animals and its resistance can be used as an indicative marker of LA-SA [13,26]. Only 18% of farm isolates were resistant to cefoxitin implying methicillin resistance. However, all these isolates were susceptible to vancomycin with the MICs ranging between 1.5 μg/mL to 3 μg/mL. Vancomycin is the drug of choice for MDR *S. aureus* infections in human health and is rarely used to treat animal infections [27]. This finding which is similar to that of a previous study that studied vancomycin susceptibility of clinical *S. aureus* show that vancomycin is still a viable treatment option of *S. aureus* infections in Zambia [28].

The isolates in the present study were more susceptible to co-trimoxazole, gentamicin and chloramphenicol ranging from 79% to 92%. Inducible resistance to macrolides, lincosamides, and group B streptogramins (MLSBi) phenotype was only detected in one isolate. MLSBi phenotype positive isolates appear to be erythromycin-resistant and clindamycin sensitive in vitro, but when given in vivo, they have constitutive *erm* mutations that render clindamycin ineffective [29]. A recent study at the largest referral hospital in Zambia found that none of the isolates had the MLSBi phenotype [28]. However, an earlier study at the same hospital reported a high rate of the MLSBi phenotype of 68.3% [19]. Many studies on *S. aureus* in animals do not report on the MLSBi phenotype probably because clindamycin is not used to treat infections in animals, however, a study from South Africa reported the MLSBi phenotype among the studied isolates from pigs [23]. Although multi-drug resistance was observed to two or more antibiotics in more than 40% of the isolates, generally our findings suggest that there are seemingly still several antibiotics that would be viable to treat infections caused by these isolates from the pig and pork production sector in Zambia.

Unexpectedly, despite the phenotypic resistance to methicillin based on resistance to oxacillin using cefoxitin disc that was detected in some of the isolates, neither the *mecA* nor *mecC* genes that encode for methicillin resistance were detected in any of the isolates. A possible explanation to the phenotypic resistance could be that the isolates are hyperproducers of penicillinases that confer some resistance to cefoxitin [30]. While the *mecA* is the mainstay gene responsible for methicillin resistance in clinical isolates, the *mecC* gene is linked to livestock associated staphylococcus especially LA-MRSA [31]. A recent study from South Africa reported the presence of the *mecC* in pig-associated *S. aureus* for the first time in Africa [32]. Relatively few countries have reported typical LA-MRSA pig-related *S. aureus* in Africa [18]. Studies from other parts of the world such as Europe and America state that intensive pig farming methods and heavy use of antibiotics are risk factors for the emergence and spread of methicillin resistance as well as resistance to other antibiotics in *S. aureus* among pigs and attending workers at farms and slaughterhouses [9,33,34]. However, none of the facilities included in the current study practises such intensive pig rearing. Markedly, MSSA cannot be overlooked as they form the reservoir from which MRSA arise [11,35]. The presence of antimicrobial resistance genes in the present study including *tet*M, *tet*K and *tet*L genes encoding for tetracycline resistance and *erm*B and *erm*C genes encoding resistance to erythromycin in some of the isolates indicate the need to closely monitor these strains as they may become a source of antimicrobial resistance given that some of these genes are harboured on plasmids which can be easily transferred between microorganisms [36].

Genes encoding the PVL and SEs were not detected in any of the isolates in the present study. While this was the first study to look for the presence of these genes in isolates from pigs in Zambia, the PVL has been reported in a previous study of clinical isolates howbeit only three out of 33 isolates were positive [37]. A study in Senegal on pigs and workers at commercial farm reported a high prevalence of the PVL gene [38]. The PVL is associated with skin and soft tissue infections and has a provenance for humans, but our study indicates that it is dispensible for pig colonisation. The role of SEs in Staphylococcal foodborne disease has been documented in several studies [39,40,41]. Therefore, the non-detection of SEs could indicate the relative safety of the pork and pork products on the Zambian market for consumers. Interestingly, several isolates harboured the IEC genes with the *sak* being the most prevalent. The staphylokinase and chemotaxis inhibitory proteins form the IEC and contribute to immune evasion in humans [12]. While IEC genes are less prevalent in livestock-adapted *S. aureus* lineages, they are considered good genetic markers for identification of human-associated *S. aureus* clones [42]. Therefore, the finding of IEC genes among *S. aureus* isolates from pigs in the present study potentiate the notion of possible anthropogenic nature of some of the *S. aureus* in Africa but could also indicate the presence of LA-SA that are well adapted to human hosts [15,18].

Our study found six *spa* types among which t1430 was the most prevalent followed by t304 mostly among *S. aureus* isolates from pigs both at the farms and abattoirs. Of significance is that t1430 and t034 are associated with CC9 and CC398 which are Livestock-associated lineages of *S. aureus* in Asia and Europe, respectively [3,4]. Therefore, our findings suggest that typical LA-SA lineages are present in pig and pork production facilities in Zambia. Generally, the *spa* types detected in pigs were different from those detected in humans in the present study, only t1430 was found in pigs and workers isolates. This would suggest distinct *S. aureus* lineages in the two populations. However, given that we could not identify the *spa* types of many isolates, we recommend further investigations into the clonal lineages using other molecular methods such as multilocus sequence typing (MLST) and whole genome sequencing (WGS) which could not be performed in the present study. Previous studies on pig related *S. aureus* isolates on the African continent are relatively few but show that the isolates have diverse *spa* types [18]. Furthermore, studies looking into the presence of LA-SA as a cause of disease among hospitalised patients in Africa are needed as this has not been reported yet but have a large impact on epidemiology of *S. aureus* infections.

## 4. Materials and Methods

### 4.1. Study Design and Sample Collection

**Figure 2:**
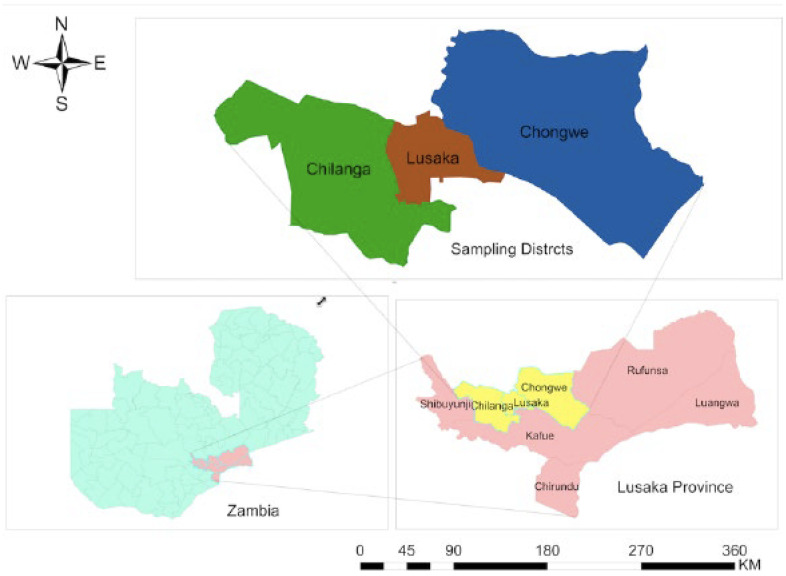
Map of the Study Sites Selected from Lusaka province in Zambia; Map was generated using the software ArcGIS version 10.3.

The study was a cross sectional study carried out between June 2020 and September 2021 in three districts of the Lusaka Province of Zambia namely Chilanga, Lusaka and Chongwe districts (Figure 2). Lusaka Province hosts many of the commercial and semi-commercial (small and medium scale rearing of pigs meant solely for selling) pig farms in Zambia. Pig farms and abattoirs in selected districts within the province were included in the study following consent from the farm and abattoir owners. The farms and abattoirs were arbitrarily grouped into three following categories based on the number of pigs at the facility: small scale (less than 100 pigs), medium scale (100 to 500 pigs) and commercial scale (greater than 500 pigs). A total of 492 pig nasal swabs were randomly collected from 13 farms and three abattoirs by inserting a swab and gently rotating it in the anterior nares. Additionally, 53 nasal and 53 hand swabs each from humans (farm workers and abattoir workers) in close contact with the pigs were collected. The human nasal swabs were collected by inserting a swab and gently rotating it in the anterior nares, whereas a hand swab was collected by gently rubbing the swab in both palms.

### 4.2. S. aureus Detection and Identification

Phenotypic detection and identification of *S. aureus* was carried out by conventional microbiological methods [43]. Briefly, each swab was transferred into 10 mL Mueller-Hinton broth (MHB) (Oxoid, Basingstoke, UK) supplemented with 6% Sodium Chloride (NaCl) and incubated at 37 °C for 16 to 20 h. Then, a loopful of the broth was inoculated on Mannitol Salt Agar (MSA) (Oxoid, Basingstoke, UK) and the plates were then incubated for 16 to 20 h at 37 °C. Resulting yellowish colonies were then inoculated onto Baird Parker Agar (BPA) (Oxoid, Basingstoke, UK) and incubated for 16 to 20 h at 37 °C. Resulting black or greyish colonies with or without a halo on the BPA plates were then grown in Brain Heart Infusion Broth (BHIB) (Oxoid, Basingstoke, UK) for 16 to 20 h at 37 °C. Using 0.5 mL of the BHIB culture, a tube coagulase test using rabbit plasma was set up according to manufacturer’s instructions (Sigma-Aldrich, Taufkirchen, Germany) by incubating the tubes at 37 °C and reading after four (4) hours and at 24 h. All coagulase positive isolates were considered as *S. aureus* and stored in 20% glycerol at −20 °C until further analysis.

### 4.3. Determination of Antimicrobial Susceptibility Profiles

The antimicrobial susceptibility of *S. aureus* to antibiotic discs (Oxoid, Basingstoke, UK) of 10 µg gentamycin, 5 µg ciprofloxacin, 15 µg erythromycin, 10 µg clindamycin, 30 µg amikacin, 10 units penicillin G, 25 µg co-trimoxazole, 30 µg chloramphenicol and 30 µg tetracycline was determined using the Kirby-Baur disc diffusion method and interpreted according to the 2020 Clinical and Laboratory Standards Institute (CLSI) guidelines [44]. Methicillin resistance was detected by checking for resistance to oxacillin using the 30 µg cefoxitin discs following the 2020 CLSI guidelines [44]. The D-test using erythromycin and clindamycin discs was also used to detect inducible resistance to macrolides, lincosamides, and group B streptogramins (MLSBi) in the *S. aureus* isolates. Susceptibility to vancomycin was determined for the isolates resistant to methicillin using vancomycin E-strips to determine the minimum inhibition concentrations (MICs) according to the CLSI guidelines [44]. Briefly, using a swab, one to two pure colonies of the organism grown overnight on nutrient agar were suspended into 2 mLs of physiological normal saline to make a 0.5 McFarland density. These bacteria were then spread evenly on a Mueller Hinton agar plate using a sterile swab. After allowing the plate to air dry for a few minutes, antimicrobial discs were gently placed on the Mueller Hinton agar plate ensuring that discs were not closer than 24 mm from centre to centre. The plates were then incubated for 16 to 24 h at 37 °C for the other antibiotics and at 35 °C for 24 h for cefoxitin. For vancomycin, one E-strip was placed per plate of the isolate, which were incubated for 16 to 24 h at 37 °C.

### 4.4. Molecular Identification and Genotyping

#### 4.4.1. DNA Extraction

Genomic DNA was prepared by thermo lysis of fresh *S. aureus* cells. Briefly, a loopful of *S. aureus* cells from Nutrient Agar (Oxoid, Basingstoke, UK) were transferred into a micro centrifuge tube containing 200 µL of 1X MiliQ water and boiled for 15 min. After cooling on ice, the DNA thermolysate were centrifuged at 14,000× *g* and then stored at −20 °C until required.

#### 4.4.2. Molecular Identification of *S. aureus*

The species identification of the isolates was then confirmed by detection of the nuclease gene using *nuc* primers (Table 6) according to a previously described PCR protocol [45].

#### 4.4.3. Detection of Methicillin Resistance Genes and Other Antimicrobial Resistance Genes

The presence of the *mecA* and *mecC* gene was checked by using previously described PCR protocols [46,47] and primers (Table 6). The erythromycin resistance encoding genes (*ermA, ermB* and *ermC*) and tetracycline resistance encoding genes (*tetK, tetL, tetM* and *tetO*) were detected using previously described protocols and primers shown in Table 6 [48,49].

#### 4.4.4. Detection of PVL and SE Genes

PCR with gene-specific primers (Table 6) was performed according to previously described protocols to detect genes encoding several virulence factors of *S. aureus* including *lukS-PV* and *lukF-PV* genes encoding PVL [47], immune evasion cluster genes (IEC) *sak*, *scn* and *chp* [12] and staphylococcal enterotoxins: *sea*, *seb*, *sec*, *sed*, and *see* [52]. PCR conditions were as described in the previous protocols, respectively [12,47,52].

#### 4.4.5. Spa Type Determination

*Spa* typing was performed using a previously described PCR protocol [51] with the primer sets shown in Table 6. Sequencing of the protein A gene (*spa*) was performed using BigDye terminator method with an ABI PRISM 3730XL DNA analyser (Applied Biosystems, Foster City, CA, USA). The DNA sequence reads were edited using the ATGC Software. The sequences obtained were then submitted to the online tool Center for Genomic Epidemiology to determine the *spa* types [53].

### 4.5. Data Analysis

Data from the study were entered into Microsoft™ excel spreadsheets, and then analysed using IBM SPSS version 25 (IBM Corp) and R. The frequencies of *S*. *aureus* in farm and abattoirs were presented as percentages and 95% confidence intervals. P values of less than 0.05 were considered statistically significant. The chromatograph sequence files of the isolates were analysed with the online tool Centre of Genomic Epidemiology (CGE) for *Spa* typing to determine the *spa* types [53].

## 5. Conclusions

The presence of *S. aureus* was high among pigs in Zambia. Furthermore, the detection of *S. aureus* on the hands and in nasal cavities of farm and abattoir workers is a public health concern. Although MRSA was only phenotypically detected, the significance of MSSA as a potential source from which MRSA can arise cannot be overlooked. The presence of plasmid mediated resistance genes and immune evasion genes among the isolates warrant continuous monitoring of *S. aureus* in this sector to combat *S. aureus* infections using a “One Health” approach in Zambia.

## Figures and Tables

**Table 1:** *S. aureus* positivity rates from pigs, humans and districts in Lusaka Province.

Factor	Category	*n* Tested	*n* Positives	Prevalence (%)	95% CI
Overall Positivity	Positive	598	198	33.1	29.4–37.1
Humans	Overall	106	12	11.3	6.2–19.3
	Hand Swabs	53	6	11.3	4.7–23.7
	Nasal Swabs	53	6	11.3	4.7–23.7
Pigs	Nasal swabs	492	186	37.8	33.5–42.3
Districts	Chongwe	250	60	24.0	18.9–29.9
	Lusaka	235	63	26.8	21.4–33.0
	Chilanga	113	75	66.4	56.6–74.8

Abbreviations: CI = Confidence interval; *n* = number of samples.

**Table 2:** Prevalence of *S. aureus* in pigs and humans at farms and abattoirs.

Study Site	Species	Type of Facility *	*n* Tested	*n* Positives	Prevalence (%)	95% CI
Farms	Combined pigs and humans	Small	53	13	24.5	14.2–38.6
		Medium	252	61	24.2	19.1–30.1
		Large	202	64	31.7	25.4–38.7
		Overall	507	138	27.2	23.4–31.3
	Pigs only	Small	45	13	28.9	16.8–44.5
		Medium	216	57	26.4	20.8–32.9
		Large	157	61	38.9	31.8–47.0
		Overall	418	131	31.3	27.0–36.1
	Humans only	Nasal	38	3	7.9	2.1–22.5
		Hand	38	4	10.5	3.4–25.7
		Overall	76	7	9.2	4.1–18.6
	Human Nasal	Small	4	0	0	0
		Medium	18	2	11.1	2.0–36.1
		Large	16	1	6.3	0.3–32.3
	Human Hand	Small	4	0	0	0
		Medium	18	2	11.1	2.0–36.1
		Large	16	2	12.5	2.2–39.6
Abattoirs	Combined pigs and humans	Medium	20	4	20	6.6–44.3
		Large	71	56	78.9	67.3–87.3
		Overall	91	60	65.9	55.2–75.3
	Pigs only	Medium	20	4	20	6.6–44.3
		Large	54	51	94.4	83.7–98.6
		Overall	71	55	77.5	65.7–86.2
	Humans **	Hand	8	2	25	4. 5–64.4
		Nasal	9	3	33.3	9.0–69.1
		Overall	17	5	29.4	11.4–56.0

* Type of facility: Small scale (less than 100 pigs), medium scale (100 to 500 pigs) and commercial scale (greater than 500 pigs; ** All human swabs from abattoirs were collected at the large facilities only. Abbreviations: CI = Confidence interval; *n* = number of samples.

**Table 3:** Antibiotic resistance patterns of *S. aureus* isolates from pigs and workers from pig farms and abattoirs in Lusaka Province.

Resistance Pattern	Proportion of Isolates % (*n*)	
	Farm Isolates (*n* = 141)	Abattoir Isolates (*n* = 63)
P	34.8 (49)	42.9 (27)
Te	1.4 (2)	1.6 (1)
P + Te	20.6 (29)	7.9 (5)
P + Cip	7.1 (10)	34.9 (22)
P + CD	0.7 (1)	3.3 (2)
P + CN + Te	2.1	1.6 (1)
P + E + TE	1.4 (2)	-
P + Te + Cip	0.7 (1)	3.2 (2)
P + E + CD + Cip	14.2 (20)	-
P + E + CD + TE	1.4 (2)	-
P + E + C + CIP	1.4 (2)	-
P + CN + TE + SXT	5.0 (7)	-
P + E + CD + TE + SXT	0.7 (1)	-
P + E + CD + CN + Cip	1.4 (2)	-
P + E + CN + Te + CIP	0.7 (1)	-
P + E + CD + CN + Te + SXT	0.7 (1)	-
^1^ Other	4.3 (6)	4.7 (3)

Abbreviations: *n* = number of samples, P = Penicillin; CN = Gentamicin; E = Erythromycin; CD = Clindamycin; Cip = Ciprofloxacin; Te = Tetracycline, SXT = Cotrimoxazole, C = Chloramphenicol, - = Not detected; ^1^ Other = P + E, P + SXT, P + E + C, P + E + CD, P + E + SXT, P + CD + Te (farm isolates) and P + Te + SXT, P + CD + CN, P + C + Cip (Abattoir isolates). Each pattern was manifested in only one isolate.

**Table 4:** IEC genes distribution among the *S. aureus* isolates (*n* = 225).

		IEC Gene		
Source (Farm or Abattoir)	Sample Type	*scn* % (*n*)	*sak*% (*n*)	*chp* % (*n)*
Farm 1	Pig nasal Swab	-	0.4 (1)	-
Farm 2	Pig nasal Swab	-	0.4 (1)	-
Farm 4	Pig nasal Swab	-	1.3 (3)	-
Farm 5	Pig nasal Swab	-	0.4 (1)	-
Farm 6	Pig nasal Swab	-	0.4 (1)	-
Farm 7	Pig nasal Swab	0.9 (2)	2.7 (6)	-
Farm 9	Pig nasal Swab	-	0.4 (1)	-
Farm 10	Pig nasal Swab	-	1.3 (3)	-
Abattoir 1	Pig nasal Swab	0.4 (1)	-	0.4 (1)
	Total	1.3 (3)	7.6 (17)	0.4 (1)

Abbreviation: *n* = number of isolates; - = None detected.

**Table 5:** *Spa* type distribution among representative farm and abattoir isolates (*n* = 43).

		*Spa* Type % (*n*)						
Species	Study Site	t1430	t034	t318	t571	t084	t899	Unknown
Humans	Farms	0	0	0	0	0	2.3 (1)	4.7 (2)
	Abattoirs	4.7 (2)	0	0	0	2.3 (1)	0	0
Pigs	Farms	14.0 (6)	9.3 (4)	9.3 (4)	2.3 (1)	0	0	25.6 (11)
	Abattoirs	9.3 (4)	9.3 (4)	0	0	0	0	7.0 (3)
	Total	28.0 (12)	18.6 (8)	9.3 (4)	2.3 (1)	2.3 (1)	2.3 (1)	37.3 (16)

Abbreviation: *n* = number of isolates.

**Table 6:** Primer sets used in the study.

Primer Name	Target Gene	Primer Sequence (5′-3′)	Amplicon Size	Reference
Nuc1	*nuc*	GCG ATT GAT GGT GAT ACG GTT	279 bp	[45]
Nuc2		AGC CAA GCC TTG ACG AAC TAA AGC		
mecA P4	*mec*A	TCCAGATTACAACTTCACCAGG	162 bp	[46]
mecA P7		CCACTTCATATCTTGTAACG		
mecA_LGA251_	*mecC*	GAAAAAAAGGCTTAGAACGCCTC	138 bp	[47]
mecA_LGA251_		GAAGATCTTTTCCGTTTTCAGC		
ermA-1	*erm*[A]	TCTAAAAAGCATGTAAAAGAA	645 bp	[48]
ermA-2		CTTCGATAGTTTATTAATATTAG		
ermB-1	*erm*[B]	GAAAAGTACTCAACCAAATA	639 bp	[48]
ermB-2		AGTAACGGTACTTAAATTGTTTA		
ermC-1	*erm*[C]	TCAAAACATAATATAGATAAA	642 bp	[48]
ermC-2		GCTAATATTGTTTAAATCGTCAAT		
tetK-1	*tet*[K]	TTAGGTGAAGGGTTAGGTCC	697 bp	[49]
tetK-2		GCAAACTCATTCCAGAAGCA		
tetM-1	*tet*[M]	GTTAAATAGTGTTCTTGGAG	576 bp	[49]
tetM-2		CTAAGATATGGCTCTAACAA		
tetL-1	*tet*[L]	CATTTGGTCTTATTGGATCG	456 bp	[49]
tetL-2		ATTACACTTCCGATTTCGG		
tetO-1	*tet*[O]	GATGGCATACAGGCACAGAC	615 bp	[49]
tetO-2		CAATATCACCAGAGCAGGCT		
pvl-FP	*lukF-PV*	GCTGGACAAAACTTCTTGGAATAT	83	[47]
pvl-RP		GATAGGACACCAATAAATTCTGGATTG		
SEA-3	*sea*	CCTTTGGAAACGGTTAAAACG	127 bp	[50]
SEA-4		TCTGAACCTTCCCATCAAAAAC		
SEB-1	*seb*	TCGCATCAAACTGACAAACG	477 bp	[50]
SEB-4		GCAGGTACTCTATAAGTGCCTGC		
SEC-3	*sec*	CTCAAGAACTAGACATAAAAGCTAGG	271 bp	[50]
SEC-4		TCAAAATCGGATTAACATTATCC		
SED-3	*sed*	CTAGTTTGGTAATATCTCCTTTAAACG	319 bp	[50]
SED-4		TTAATGCTATATCTTATAGGGTAAACATC		
SEE-3	*see*	CAGTACCTATAGATAAAGTTAAAACAAGC	178 bp	[50]
SEE-2		TAACTTACCGTGGACCCTTC		
Sak-1	*sak*	AAGGCGATGACGCGAGTTAT	223 bp	[12]
Sak-2		GCGCTTGGATCTAATTCAAC		
Chp-1	*chp*	GAAAAAGAAATTAGCAACAACAG	410 bp	[12]
Chp-2		CATAAGATGATTTAGACTCTCC		
Scn-1	*scn*	AGCACAAGCTTGCCAACATCG	258 bp	[12]
Scn-2		TTAATATTTACTTTTTAGTGC		
1095F	*spa*	AGACGATCCTTCGGTGAGC	variable	[51]
1517R		GCTTTTGCAATGTCATTTACTG		

## Data Availability

Sequences for the *spa* types have deposited in the DDBJ.

## Supplementary Materials

The following supporting information can be downloaded at: https://www.mdpi.com/article/10.3390/antibiotics11070844/s1, Table S1: Samples for which more than one *S. aureus* colony type was isolated; Table S2: Vancomycin MICs of *S. aureus*; Table S3: Characteristics of *S. aureus* Isolates Sequenced for *Spa* typing. Reference [44] is cited in the supplementary materials.
