# Supplementary material for: Antimicrobial Susceptibility Profiles and Molecular Characterisation of Staphylococcus aureus from Pigs and Workers at Farms and Abattoirs in Zambia
Source: Antibiotics (Basel). 2022 Jun 24;11(7):844. doi: 10.3390/antibiotics11070844 (PMC9311834; doi:10.3390/antibiotics11070844)
Supplement: Supplementary file 1 [file antibiotics-11-00844-s001.zip › Revised Suppiementary Table S1_Samples for which more than one S. aureus colony type was isolated.pdf]

Table S1: Samples for which more than one *S. aureus* colony type was isolated

Study No.	District	Study Site	Type of Facility	Species	Sample Type	S.aureus Isolation
P4-4-1	Lusaka	Farm 4	Small Scale	Pig	Pig nasal swab	Yes
P4-8-1	Lusaka	Farm 4	Small Scale	Pig	Pig nasal swab	Yes
P5-14-1	Lusaka	Farm 5	Small Scale	Pig	Pig nasal swab	Yes
P6-8-1	Chongwe	Farm 6	Medium Scale	Pig	Pig nasal swab	Yes
P7-3-1	Chongwe	Farm 7	Medium Scale	Pig	Pig nasal swab	Yes
P10-4-1	Chongwe	Farm 10	Medium Scale	Pig	Pig nasal swab	Yes
P10-10-1-1-2	Chongwe	Farm 10	Medium Scale	Pig	Pig nasal swab	Yes
P10-10-1	Chongwe	Farm 10	Medium Scale	Pig	Pig nasal swab	Yes
P10-7-1	Chongwe	Farm 10	Medium Scale	Pig	Pig nasal swab	Yes
P10-6-1	Chongwe	Farm 10	Medium Scale	Pig	Pig nasal swab	Yes
P10-17-1	Chongwe	Farm 10	Medium Scale	Pig	Pig nasal swab	Yes
P10-23-1	Chongwe	Farm 10	Medium Scale	Pig	Pig nasal swab	Yes
P10-26-1	Chongwe	Farm 10	Medium Scale	Pig	Pig nasal swab	Yes
P10-9-1	Chongwe	Farm 10	Medium Scale	Pig	Pig nasal swab	Yes
P10-26-2	Chongwe	Farm 10	Medium Scale	Pig	Pig nasal swab	Yes
A1-6-1	Chilanga	Abattoir 1	Large Scale	Pig	Pig nasal swab	Yes
A1-7-1	Chilanga	Abattoir 1	Large Scale	Pig	Pig nasal swab	Yes
A1-12-1	Chilanga	Abattoir 1	Large Scale	Pig	Pig nasal swab	Yes
A1-13-1	Chilanga	Abattoir 1	Large Scale	Pig	Pig nasal swab	Yes
A1-22-1	Chilanga	Abattoir 1	Large Scale	Pig	Pig nasal swab	Yes
AH1-1-1	Chilanga	Abattoir 1	Large Scale	Pig	Pig nasal swab	Yes
P11-20-1	Lusaka	Farm 11	Large Scale	Pig	Pig nasal swab	Yes
P11-42-1	Lusaka	Farm 11	Large Scale	Pig	Pig nasal swab	Yes
P11-51-1	Lusaka	Farm 11	Large Scale	Pig	Pig nasal swab	Yes
P12-13-1	Lusaka	Farm 12	Large Scale	Pig	Pig nasal swab	Yes
P13-14-1	Lusaka	Farm 13	Large Scale	Pig	Pig nasal swab	Yes
P13-16-1	Lusaka	Farm 13	Large Scale	Pig	Pig nasal swab	Yes
