# Supplementary material for: Antimicrobial Susceptibility Profiles and Molecular Characterisation of Staphylococcus aureus from Pigs and Workers at Farms and Abattoirs in Zambia
Source: Antibiotics (Basel). 2022 Jun 24;11(7):844. doi: 10.3390/antibiotics11070844 (PMC9311834; doi:10.3390/antibiotics11070844)
Supplement: Supplementary file 1 [file antibiotics-11-00844-s001.zip › Revised Supplementary Table S3_Characteristics of S. aureus Isolates Sequenced for Spa typing.pdf]

Table S3: Characteristics of *S. aureus* Isolates Sequenced for *Spa* typing

Spa type	Isolate ID	Sample type	Study site	Type of Facility*	District	Resistance Phenotype
t1430	P3-10	Pig nasal swab	Farm 3	Large scale	Chongwe	P+E+CD+Cip
t1430	P3-23	Pig nasal swab	Farm 3	Large scale	Chongwe	P+Cip
t1430	P12-30	Pig nasal swab	Farm 12	Large scale	Lusaka	P+E+C+Cip
t1430	P10-12	Pig nasal swab	Farm 10	Medium scale	Chongwe	P+Cip
t1430	AH1-4	Human hand swab	Abattoir 1	Large scale	Chilanga	P+Cip
t1430	A1-13-1	Pig nasal swab	Abattoir 1	Large scale	Chilanga	P+Cip
t1430	A1-10	Pig nasal swab	Abattoir 1	Large scale	Chilanga	P+C+Cip
t1430	A1-1	Pig nasal swab	Abattoir 1	Large scale	Chilanga	P+Cip
t1430	P10-4-1	Pig nasal swab	Farm 10	Medium scale	Chongwe	P+Cip
t1430	P10-9	Pig nasal swab	Farm 10	Medium scale	Chongwe	P
t1430	A1-2	Pig nasal swab	Abattoir 1	Large scale	Chilanga	P+Cip
t1430	AH1-1-1	Human hand swab	Abattoir 1	Large scale	Chilanga	P+Te+Cip
t034	P13-25	Pig nasal swab	Farm 13	Large scale	Lusaka	CX+P+E+Te
t034	A2-11	Pig nasal swab	Abattoir 2	Large scale	Chilanga	P+Te+SXT
t034	A2-10	Pig nasal swab	Abattoir 2	Large scale	Chilanga	P+Te+SXT
t034	P7-39	Pig nasal swab	Farm 7	Medium scale	Chongwe	Te
t034	A3-2	Pig nasal swab	Abattoir 3	Medium scale	Lusaka	Te
t034	A2-6	Pig nasal swab	Farm 2	Large scale	Chongwe	P+Te
t034	A3-6	Pig nasal swab	Abattoir 3	Medium scale	Lusaka	P+Te
t318	P2-2	Pig nasal swab	Farm 2	Medium scale	Chilanga	P
t318	P6-28	Pig nasal swab	Farm 6	Medium scale	Chongwe	P
t318	P7-3	Pig nasal swab	Farm 7	Medium scale	Chongwe	P+Te
t318	P7-6	Pig nasal swab	Farm 7	Medium scale	Chongwe	P+Te
t318	P4-12	Pig nasal swab	Farm 4	Medium scale	Lusaka	Susceptible to all
t084	A2H-10	Human nasal swab	Abattoir 2	Large scale	Chilanga	P+Te
t571	P1-8	Pig nasal swab	Farm 1	Medium scale	Chilanga	P+Te
t899	H6-4	Human nasal swab	Farm 6	Medium scale	Chongwe	P
Unknown	P4-4	Pig nasal swab	Farm 4	Medium scale	Lusaka	P
Unknown	P4-8	Pig nasal swab	Farm 4	Medium scale	Lusaka	P
Unknown	P11-48	Pig nasal swab	Farm 11	Large scale	Lusaka	CX+P
Unknown	P7-1	Pig nasal swab	Farm 7	Medium scale	Chongwe	P+Te
Unknown	H1-2	Human hand swab	Farm 1	Medium scale	Chilanga	P+Te
Unknown	H3-13	Human hand swab	Farm 3	Medium scale	Chongwe	P+E+CD+Cip
Unknown	P7-4	Pig nasal swab	Farm 7	Medium scale	Chongwe	P
Unknown	P1-5	Pig nasal swab	Farm 1	Medium scale	Chilanga	P
Unknown	P4-2	Pig nasal swab	Farm 4	Medium scale	Lusaka	P
Unknown	P4-1	Pig nasal swab	Farm 4	Medium scale	Lusaka	P
Unknown	A2-13	Pig nasal swab	Abattoir 2	Large scale	Chilanga	P
Unknown	A2-18	Pig nasal swab	Abattoir 2	Large scale	Chilanga	P
Unknown	A2-19	Pig nasal swab	Abattoir 2	Large scale	Chilanga	P
Unknown	P5-18	Pig nasal swab	Farm 5	Medium scale	Lusaka	P
Unknown	P7-33	Pig nasal swab	Farm 7	Medium scale	Chongwe	P+Te+Cip
Unknown	P10-10-1	Pig nasal swab	Farm 10	Medium scale	Chongwe	P+CD+Te

*Type of facility: Small scale (less than 100 pigs), medium scale (100 to 500 pigs) and commercial scale (greater than 500 pigs;
