# Supplementary material for: Antimicrobial Susceptibility Profiles and Molecular Characterisation of Staphylococcus aureus from Pigs and Workers at Farms and Abattoirs in Zambia
Source: Antibiotics (Basel). 2022 Jun 24;11(7):844. doi: 10.3390/antibiotics11070844 (PMC9311834; doi:10.3390/antibiotics11070844)
Supplement: Supplementary file 1 [file antibiotics-11-00844-s001.zip › Supplementary Table S2_Vancomycin MIC Results.pdf]

Table S2: Vancomycin MICs of *S. aureus**

Isolate ID	Sample Type	Study Site	District	Vancomycin MIC (μg/mL)	Susceptibility**
P11-16	Pig nasal swab	Farm 11	Lusaka	1.5	Susceptible
P11-17	Pig nasal swab	Farm 11	Lusaka	1.5	Susceptible
P11-18	Pig nasal swab	Farm 11	Lusaka	1.5	Susceptible
P11-22-1	Pig nasal swab	Farm 11	Lusaka	1.5	Susceptible
P11-26	Pig nasal swab	Farm 11	Lusaka	1.5	Susceptible
P11-27	Pig nasal swab	Farm 11	Lusaka	1.5	Susceptible
P11-30	Pig nasal swab	Farm 11	Lusaka	1.5	Susceptible
P11-39	Pig nasal swab	Farm 11	Lusaka	1.5	Susceptible
P11-42-1	Pig nasal swab	Farm 11	Lusaka	1.5	Susceptible
P11-43	Pig nasal swab	Farm 11	Lusaka	1.5	Susceptible
P11-46	Pig nasal swab	Farm 11	Lusaka	1.5	Susceptible
P11-46	Pig nasal swab	Farm 11	Lusaka	1.5	Susceptible
P11-46-1	Pig nasal swab	Farm 11	Lusaka	1.5	Susceptible
P11-50	Pig nasal swab	Farm 11	Lusaka	2.0	Susceptible
P11-51	Pig nasal swab	Farm 11	Lusaka	1.5	Susceptible
P11-51-1	Pig nasal swab	Farm 11	Lusaka	1.5	Susceptible
P13-1	Pig nasal swab	Farm 13	Lusaka	1.5	Susceptible
P13-2	Pig nasal swab	Farm 13	Lusaka	2.0	Susceptible
P13-12	Pig nasal swab	Farm 13	Lusaka	1.5	Susceptible
P13-16	Pig nasal swab	Farm 13	Lusaka	1.5	Susceptible
P13-25	Pig nasal swab	Farm 13	Lusaka	1.5	Susceptible
P13-33	Pig nasal swab	Farm 13	Lusaka	3.0	Susceptible
P13-34	Pig nasal swab	Farm 13	Lusaka	1.5	Susceptible
P13-50	Pig nasal swab	Farm 13	Lusaka	1.5	Susceptible
P13-51	Pig nasal swab	Farm 13	Lusaka	1.5	Susceptible

*All isolates tested were resistant to cefoxitin; ** Susceptibility was interpreted according to the 2020 Clinical and Laboratory Standards Institute (CLSI) guidelines [1]

References

1 Clinical and Laboratory Standards Institute. Performance Standards for Antimicrobial Susceptibility Testing.30th ed. CLSI supplement M1002020.
